# Precise calcium-to-spike inference using biophysical generative models

**DOI:** 10.21203/rs.3.rs-6017950/v1

**Published:** 2025-04-24

**Authors:** Gerard Joey Broussard, Giovanni Diana, Francisco J. Urra Quiroz, B. Semihcan Sermet, Nelson Rebola, Laura A. Lynch, David A. DiGregorio, Samuel S.-H. Wang

**Affiliations:** 1 Neuroscience Institute, Washington Road, Princeton University, Princeton, New Jersey USA 08540.; 2 Institut Pasteur, Université Paris Cité, CNRS UMR 3571, Synapse and Circuit Dynamics Laboratory, Paris, France.; 3 L’Institut du Cerveau et de la Moelle épinière, CNRS UMR 7225, Paris, France; 4 Department of Physiology and Biophysics, University of Colorado School of Medicine, Aurora, Colorado USA 80045.

## Abstract

The intramolecular dynamics of fluorescent indicators of neural activity can distort the accurate estimate of action potential (“spike”) times. In order to develop a more accurate spike inference algorithm we characterized the kinetic responses to calcium of three popular indicator proteins, GCaMP6f, jGCaMP7f, and jGCaMP8f, using in vitro stopped-flow and brain slice recordings. jGCaMP8f showed a use-dependent slowing of fluorescence responses that caused existing inference methods to generate numerous false positives. From these data we developed a multistate model of GCaMP and used it to create Bayesian Sequential Monte Carlo (Biophys_SMC_) and machine learning (Biophys_ML_) inference methods that reduced false positives substantially. This biophysical method dramatically improved spike time accuracy, detecting individual spikes with a median uncertainty of 4 milliseconds, a performance level that reached the theoretical limit and is twice as accurate as any previous method. Our framework thus highlights advantages of physical model-based approaches over model-free algorithms.

## Introduction

Genetically encoded calcium indicators (GECIs), as exemplified by the GCaMP family of sensors (Tian et al., 2009; Akerboom et al., 2012; Chen et al., 2013; Dana et al., 2019; Zhang et al., 2023), have transformed the study of neural populations by enabling simultaneous recordings from large ensembles of neurons (Demas et al., 2021). These sensors convert rapid changes in intracellular calcium concentration, which accompany neuronal action potentials, into measurable fluorescence signals. However, the temporal resolution of GECIs is limited by their slower kinetics compared to the rapid voltage changes associated with neuronal spikes. This mismatch limits the temporal precision with which the neural dynamics underlying behavior can be accurately estimated (Xiao et al., 2017; Li et al., 2019; Wei et al., 2020).

A variety of computational strategies have been developed to address the challenge of inferring spikes from fluorescence signals. These methods can be broadly categorized as either supervised or unsupervised. Supervised approaches leverage training data where both fluorescence and spike times are known to train machine learning models to recognize statistical regularities mapping observed signals to underlying spiking activity (Rupprecht et al., 2021; Zhou et al., 2023). Unsupervised approaches, by contrast, do not require labeled data and instead use principled assumptions about how spikes generate fluorescence based on the statistical properties of fluorescence time series to infer the underlying “latent” spike trains (e.g. Vogelstein et al., 2010; Pnevmatikakis et al., 2013; Deneux et al., 2016; Friedrich et al., 2017; Sebastian et al., 2017; Pachitariu et al., 2018). The performance of both categories is enhanced if the relationship between spike occurrences and the resultant fluorescence responses is fundamentally linear. When this linearity assumption holds, unsupervised methods can utilize simple, computationally efficient models (in particular linear deconvolution; Friedrich et al., 2017), while supervised methods benefit from stable, direct mappings that allow for robust training and generalization.

However, the spike-to-fluorescence relationship often deviates from the linear ideal. Classic examples include saturation of indicator fluorescence at high calcium concentrations (Regehr and Atluri, 1995) and slowing of calcium dynamics due to calcium buffering by high-affinity indicators (Sabatini and Regehr, 1998). Thirdly, GECI responses to free calcium changes can be supralinear (Rose et al., 2014). Such nonlinearities pose potential challenges to both unsupervised and supervised methods. Popular unsupervised methods relying on low-complexity models such as linear convolution or generative frameworks reliant upon a static decay process would be expected to exhibit systematic errors dictated by the nature of the nonlinearity. Supervised methods, meanwhile, may fail to capture the complexity of novel, nonlinear regimes that differ substantially from the conditions under which they were trained (Seroussi and Zeitouni, 2022; Sariyildiz et al., 2023). Consequently, nonlinearities in the spike-to-fluorescence transformation have the potential to degrade the accuracy and reliability of inferred spike statistics.

The recently developed jGCaMP8 sensors represent a promising advance due to their faster rise times and improved single-spike signal-to-noise ratios (SNR) (Zhang et al., 2023; Diana et al., 2024), but little is known about what new non-linearities they introduce. Past generation indicators have been reported to exhibit supralinear responses arising from closely occurring spikes (Chen et al., 2013; Éltes et al., 2019; Wei et al., 2020; Huang et al., 2021; Schoenfeld et al., 2021). Neither models designed to capture this supralinearity (Deneux et al., 2016; Wei et al., 2020) nor models that assume linear characteristics of the newer probes (Zhang et al., 2023) are able to extract more accurate spiking dynamics using jGCaMP8 than they do from previous-generation sensors.

To address these gaps, we first comprehensively characterized the dynamics of GCaMP6f, jGCaMP7f, and jGCaMP8f responses. We identified novel nonlinear properties of these responses, including a pronounced use-dependent slowing by jGCaMP8f during periods of heightened activity. We developed a biophysical model of all three GCaMPs, which we used as the core of two new spike inference approaches: a sequential Monte Carlo (SMC) method to model latent spike-time sequences, and a machine learning-based strategy trained on synthetic data generated by the SMC method. Both approaches identify spike times with higher accuracy and temporal precision than any currently used technique.

## Results

### Biophysical characterization of GCaMPs

**Figure 1:**
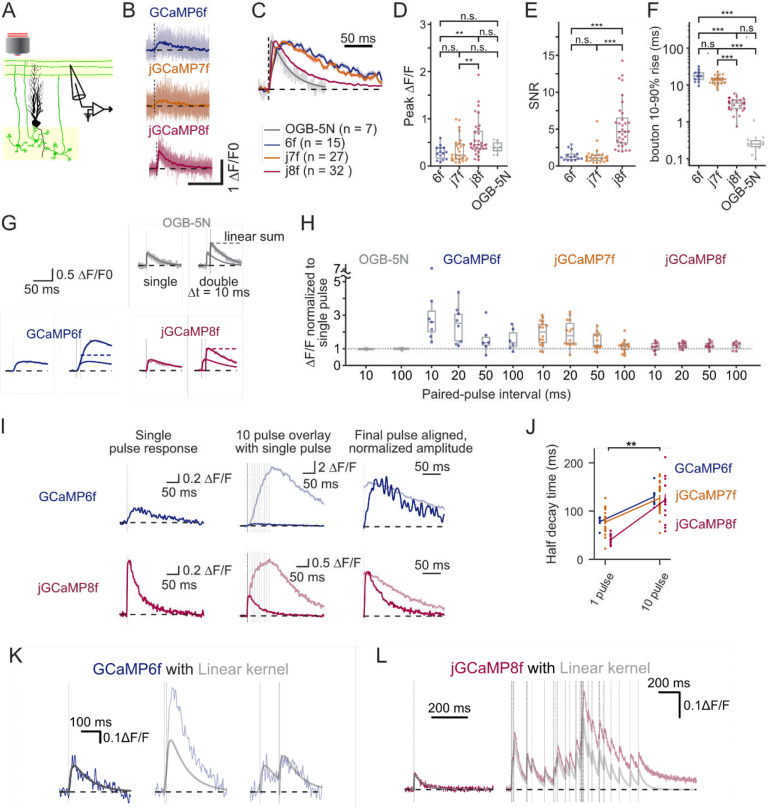
Nonlinear response characteristics of fast GCaMPs in a controlled, ex vivo setting. (**A**) Schematic of experimental approach showing sites of extracellular stimulation and imaged regions (highlighted in yellow). (**B**) Representative traces obtained across sensors for single-spike responses, with mean overlaid. (**C**) Traces from **B** averaged and normalized to compare kinetic properties of GCaMPs with the low affinity synthetic indicator, OGB-5N. (**D-F**) Peak response amplitude, signal-to-noise ratio, and 10–90% rise time for individual boutons. Each symbol represents an average across trials in one bouton. (**G**) averaged responses to one stimulus or two stimuli spaced 10 ms apart. (**H**) Relative amplitude of responses to a second stimulus spaced at various time intervals from the first stimulus. Each point represents the average response for one bouton. (**I**) Responses to 1 stimulus (left), 10 stimuli at 100 Hz overlaid to the single stimulus response (center), and normalized to the amplitude of a single-stimulus response to demonstrate prolonged decay (right). (**J**) Half-decay times as a function of stimulus number. Error bars indicate standard error of the mean. (**K-L**) Convolution of a single-stimulus response with the times of stimuli to demonstrate effect of short-term amplitude non-linearity in GCaMP6f responses in **K** and cumulative signal arising from many stimuli for jGCaMP8f in **L**. Stars indicate statistical significance as: *p<0.05, **p<0.01, ***p<0.001.

jGCaMP8 variants have been reported to possess improved kinetic properties and linear responses to action potentials compared to earlier GCaMP versions (Zhang et al., 2023). To precisely quantify these improvements, we expressed GCaMP6f, jGCaMP7f, or jGCaMP8f in cerebellar granule cells using adeno-associated viral vectors. We performed high-speed (0.5–1 kHz) two-photon linescans to measure fluorescence transients in single boutons (parallel fibers, PFs; Figure 1A–C) in response to extracellularly-evoked action potentials. jGCaMP8f exhibited the fastest rise and decay times (Figure 1C), and a fractional per-spike fluorescence change of 0.59 ± 0.07 (*n* = 32 boutons, Figure 1D), larger than the other probes tested (GCaMP6f, 0.29 ± 0.04, *n* = 14 boutons; jGCaMP7f, 0.34 ± 0.05, *n* = 27; the small-molecule indicator Oregon Green BAPTA-5N, OGB-5N, 0.39 ± 0.04, *n* = 11; mean ± SEM; ANOVA, *p* = 0.0041). jGCaMP8f showed a signal-to-noise ratio of 5.6 ± 0.1 (Figure 1E), again higher than the other probes (GCaMP6f, 1.4 ± 0.2; jGCaMP7f, 1.3 ± 0.2; jGCaMP8f, 5.6 ± 0.1; mean ± SEM; ANOVA, *p* = 6.7e-10).

The rise time of jGCaMP8f was 3.1 ± 0.2 ms, approaching that of OGB-5N (0.3 ± 0.1 ms), which has near diffusion-limited calcium on-binding kinetics (DiGregorio et al., 1999) (Figure 1F) and was faster than all other probes (GCaMP6f, 18.1 ± 1.6 ms; jGCaMP7f, 13.7 ± 0.7 ms;; mean ± SEM; ANOVA, *p* = 6.7e-29; significant Tukey HSD post hoc comparisons: *p* = 1.1e-21, GCaMP6f vs. OGB5N; *p* = 5.4e-4, jGCaMP6f vs. jGCaMP7f; *p* = 3.9e-23, jGCaMP6f vs. jGCaMP8f; *p* = 8.7e-18, jGCaMP7f vs. OGB-5N; *p* = 1.5e-19, jGCaMP7f vs. jGCaMP8f).

Recent reports of GCaMP6 indicators suggest a nonlinear relationship between the number of APs and the corresponding fluorescence response (Wei et al., 2020; Huang et al., 2021; Zhang et al., 2023). To quantify the time-dependence of this nonlinearity across fast indicator variants, we explored signals evoked by pairs of spikes delivered at defined time intervals. GCaMP6f and GCaMP7f showed short-term supralinearity that decreased to a linear summation for intervals greater than 100 ms (Figure 1G, H) (GCaMP6f, 3.1 ± 0.7 at 10 ms and 1.5 ± 0.3 at 100 ms fold increase, *n* = 7 boutons; jGCaMP7f, 1.9 ± 0.2 at 10 ms and 1.1 ± 0.1 at 100 ms, *n* = 14 boutons; mean ± SEM; two-way mixed ANOVA, *F*(3,60) = 7.2, *p* = 3.3e-4 main effect of indicator, with significant one-way ANOVA at 10 ms, F(3,32) = 6.4, *p* = 0.0016; significant Tukey HSD post hoc comparisons: *p* = 0.01, GCaMP6f vs. OGB-5N). In contrast, the jGCaMP8f responses were near-linear in their response amplitude to spikes at all tested intervals (Figure 1G, H) (testing against a null distribution centered on 1; one-sample t-test, *p* = 0.32).

To examine responses to bursts of activity, we measured bouton fluorescence in response to 1, 2, and 10 AP stimuli delivered at 100 Hz. GCaMP6f responses showed a strong supralinearity, in which a 10-pulse train evoked signals approximately 20-fold larger than a single pulse (Figure 1G, top), consistent with positive cooperativity. In contrast, jGCaMP8f responses under the same comparison resulted in only a 3-fold larger response (Figure 1F, bottom). These findings were broadly consistent with indicator saturation and low apparent cooperativity (Figure 1F, bottom).

Unexpectedly, all three tested GCaMPs showed a slowing of the decay of fluorescence transients driven by ten spikes compared with a single spike (Figure 1I) (GCaMP6f, 78 ± 5 ms 1 pulse vs. 130 ± 9 ms 10 pulse, *n* = 6 boutons; jGCaMP7f, 77 ± 5 ms 1 pulse vs. 126 ± 7 ms 10 pulse, *n* = 22 boutons; jGCaMP8f, 41 ± 3 ms 1 pulse vs. 123 ± 12 ms 10 pulse, *n* = 14 boutons; mean ± SEM; two-way mixed ANOVA, *F*(1,78) = 72.2, *p* = 1.0e-12 main effect of time). This use-dependent effect was particularly pronounced for jGCaMP8f (~3-fold slowing) when compared to the older fast sensor variants (~1.5-fold slowing) (Figure 1J, K). Further analysis revealed that a two-component exponential was required to achieve acceptable fits to the decay even for single-spike responses. Both kinetic components tended to increase in duration with the number of spikes (Supplementary Figure 1).

To explore the impact of nonlinear properties of the old and new GCaMP variants, we compared experimentally-measured spike responses to a linear model. The model was calculated by convolving a kernel fitted to a single spike response with the spike train used to generate the observed data. In the paired-spike protocol, the GCaMP6f kernel underestimated the response to a 10 ms inter-spike interval. The estimate improved as the intervals lengthened and the nonlinearity decreased (Figure 1J). When jGCaMP8f boutons were exposed to a 10 Hz Poisson stimulus train, the kernel estimate diverged increasingly from the real data (Figure 1K). Overall, these analyses reveal that the GCaMP6f amplitude nonlinearity is dependent on spike timing, while the use-dependent slowing of jGCaMP8f signals is dependent on spike rate. These findings indicate activity regimes where linear methods for deconvolution are expected to be unsuccessful: short inter-spike intervals for older GCaMPs, and high spike rates for jGCaMP8f.

**Figure 2:**
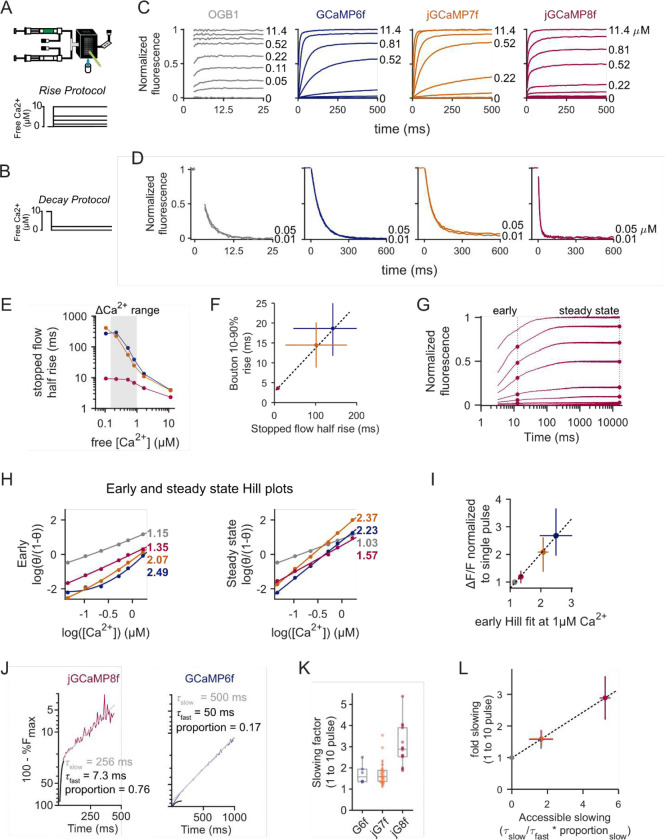
Stopped-flow fluorimetry of GCaMP dynamic responses. (**A**) Schematic of stopped-flow experiment and rising steps of calcium concentration. (**B**) Step-down protocols for decreasing calcium. (**C**) Responses to steps up from 0 μM free calcium. (**D**) Responses to steps down from 10 μM free calcium. (**E**) Half-rise times for GCaMP6f, jGCaMP7f, and jGCaMP8f as a function of calcium step size. Shaded gray region indicates the physiological range of intracellular signals. (**F**) Comparison of brain slice presynaptic bouton signals with stopped-flow rise times for the three indicator variants (**G**) Rising phase responses on logarithmic time scales. Symbols indicate the time points plotted in (**H**). (**H**) Hill plots of rising and steady-state responses to steps in calcium. Colored numbers indicate Hill coefficients. (**I**) Comparison of brain slice amplitude nonlinearity at 10 ms inter-stimulus response with respect to Hill coefficients. (**J**) Representative stopped-flow traces with y-axis values presented on a log scale for jGCaMP8f (left) and GCaMP6f (right). Sums of exponentials for each indicator demonstrate the biphasic response and the greater contrast in speed of kinetic components seen in jGCaMP8f. (**K**) Quantification of the factor of slowing seen in GCaMP fluorescence decay in response to 1 and 10 stimuli in brain slices. (**L**) Comparison of brain slice slowing to accessible slowing (see main text and methods). Error bars in all panels represent the interquartile range of the distribution.

To gain insight into the mechanistic basis for GCaMP’s use-dependent nonlinearities, we measured GCaMP fluorescence responses to various calcium concentration steps directly using stopped-flow fluorimetry on purified protein (Figure 2A, B). We first optimized stopped-flow buffer conditions to closely match intracellular ionic composition, including magnesium, which we found to be necessary to reproduce the kinetics and dynamic range of the GCaMP responses observed in living cells (Supplementary Figure 2, 3). The rise and decay kinetics of jGCaMP8f were faster than 6f and 7f for all calcium concentration steps in the range of 0–10 μM (Figure 2C, D). Within the range of calcium increase expected in response to a small number of spikes (shaded region, Figure 2E), the rise time of spike-evoked calcium transients for each GECI variant correlated with stopped-flow rise times (Figure 2E, F). Similarly, the paired-pulse facilitation of a GCaMP’s fluorescent response in neurons closely tracked cooperativity measures derived from analysis of the early stopped-flow response (Figure 2G–I). These experiments show that our stopped-flow measurements capture key features of action potential-evoked calcium responses in neurons.

Previous studies showed that activation of GCaMPs by calcium occurs via two pathways, one fast and one slow (Sun et al., 2013; Badura et al., 2014). Closer inspection of the binding and unbinding kinetics across GCaMP sensors revealed fast and slow phases of fluorescence increases. Responses of GCaMP6f and jGCaMP7f arose predominantly from a slow component whose speed increased with the size of the calcium step. In contrast, jGCaMP8f’s fluorescence developed predominantly through a fast component. While both kinetic components contributed to fluorescence changes in all GECIs, jGCaMP8f was distinguished by having a faster and larger contribution from the fast component at all concentrations tested (Figure 2J, Supplementary Figure 4).

The presence of two time constants in the rising phase of the fluorescence response is consistent with entrance into at least two distinct fluorescence states. The presence of multiple fluorescence states could account for fast and slow decay rates. We compared the time constants governing this fast and slow increase to the use-dependent slowing effect observed in our ex vivo (i.e. brain slice) recordings. We found a linear relationship between our ex vivo observations and a metric we term accessible slowing which represents a tradeoff between the speed of entrance into the fast versus slow state and stability of the slow state (Figure 2K, L). These observations support the conclusion that large calcium increases push GCaMPs into a stable fluorescent state from which they are slow to exit.

### Development of a biophysical model of GCaMP

**Figure 3:**
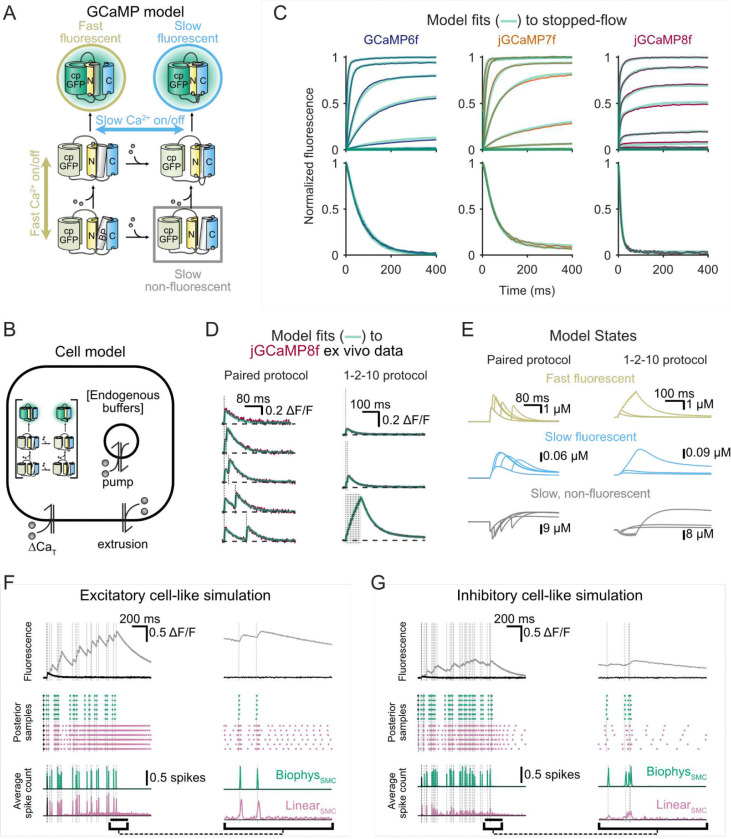
A biophysical model for GCaMP response. (**A**) Schematic GCaMP model depicting probe components, including circularly permuted green fluorescent protein (cp-GFP), the N- and C-lobes of the calcium binding-calmodulin, and the binding peptide (BP). Black arrows indicate key pathways to fluorescence with states highlighted for their role in model function. (**B**) Schematic diagram of physiological response of a cellular structure containing endogenous calcium buffer, GCaMP, and intracellular and plasma membrane calcium extrusion (pump) mechanisms. (**C**) Fits of the GCaMP model to the three GCaMP variants with different parameters for each variant. (**D**) Fits of the cell model to physiological data with fixed GCaMP model parameters. Vertical lines indicate action potential (spike) times. (**E**) Occupancy of the fast-fluorescent, slow-fluorescent, and slow non-fluorescent states of GCaMP indicating a mechanism for use-dependent slowing of fluorescence decay, becoming most apparent after 10 action potentials. (**F**) Predictions on simulated ground-truth data for an excitatory neuron. The data consists of a single spike (black) or a 10 Hz Poisson train of spikes (gray) (top panel). Lower panels show the average posterior spike probability (solid lines) and spikes times from a single trajectory (triangles) derived by our SMC method employing either the biophysical model (Biophys_SMC_, green) or a linear model (Linear_SMC_, pink) as its generative engine. Vertical lines indicate action potential (spike) times. (**G**) As in (**F**) with simulated ground truth for an inhibitory cell (30 Hz Poisson spike rate, calcium increase per spike reduced by 2, dynamic range reduced by a factor of 2).

We built a biophysical model of GCaMP that included parallel fast and slow kinetic pathways to multiple fluorescence states. Multiple kinetic pathways are known to coexist in calmodulin, the calcium-sensing domain of GCaMP, which semi-independently binds calcium via its N-lobe (fast-binding) and C-lobe (slow-binding) (Linse et al., 1991; Faas et al., 2011), each subsequently capable of interacting with a target peptide to induce fluorescence (Johnson et al., 1996; Brown et al., 1997; Sun et al., 2013; Lai et al., 2015). We reasoned that such a model structure might phenomenologically account for both the *in vitro* and *ex vivo* observations (Figure 3A). In order to estimate the kinetic rate constants of the GCaMP model we fit it to experimental data. We took advantage of a neuron type (cerebellar granule cells) with well-characterized calcium buffering and handling (Rebola et al., 2019). The cell model used here included endogenous buffering, calcium extrusion mechanisms, and calcium exchange from intracellular stores (Figure 3B) and could reproduce the fast dynamics of previous buffer models (Rebola et al., 2019), but we also added a second compartment to account for a slow decay that was not used to constrain previous models. By jointly fitting the stopped-flow data and *ex vivo* responses, we derived the final GCaMP model parameters for each variant, which were then fixed for all subsequent analyses (Supplementary Figure 5; Supplementary Figure 6A, B). This approach allowed us to reproduce observed dynamics from all experimental systems and GCaMP variants, despite the idiosyncrasies observed across the sensors (Figure 3C, D; Supplementary Figure 6C–F).

Further analyses of the GCaMP model states (Figure 3A; Supplementary Figure 6A) revealed additional insights into how parameterization led to different operational modes. When separating the fluorescent GCaMP model states into those that depended on C-lobe binding (slow fluorescent) and those that did not (fast fluorescent), we observed that jGCaMP8f alone showed stable occupancy of the fast fluorescent states in both stopped-flow and ex vivo fits (yellow traces Supplementary Figure 6C–F). The slowing of jGCaMP8f responses following spike bursts, in contrast, was driven by increased occupancy of a slow, fluorescent and non-fluorescent state (Figure 3E, blue and gray, respectively).

### Model-based insights enable improved methods of spike detection

We next leveraged the GCaMP model to implement a generative spike inference algorithm. Inspired by recent successes of a Bayesian autoregressive-model based approach (Diana et al. 2024), we adopted the same Sequential Monte Carlo (SMC) framework in which time-invariant parameters (such as those governing cell calcium handling) and latent states (such as spike times) are jointly estimated in an iterative manner to estimate spike times (Supplementary Figure 7).

As an initial test of this approach, we generated ground truth simulations using the GCaMP model and attempted inference using our SMC framework with either the GCaMP model (Biophys_SMC_ hereafter) or a linear autoregressive model (Linear_SMC_) as the generative model (Figure 3F, G).Using this approach, Linear_SMC_ produced a progressive increase in the number of false spike predictions during periods of elevated spiking for both cell types, particularly in the decay phase of the fluorescence. This is likely due to the use-dependent slowing of jGCaMP8f fluorescence. In contrast, Biophys_SMC_, which accommodated all nonlinearities, made consistent, accurate predictions of spike times across the simulation epoch. The Biophys_SMC_ was able to reproduce the input spike train with higher accuracy and precision in the context of simulations designed to mimic different cell types, including both excitatory and inhibitory cells.

**Figure 4:**
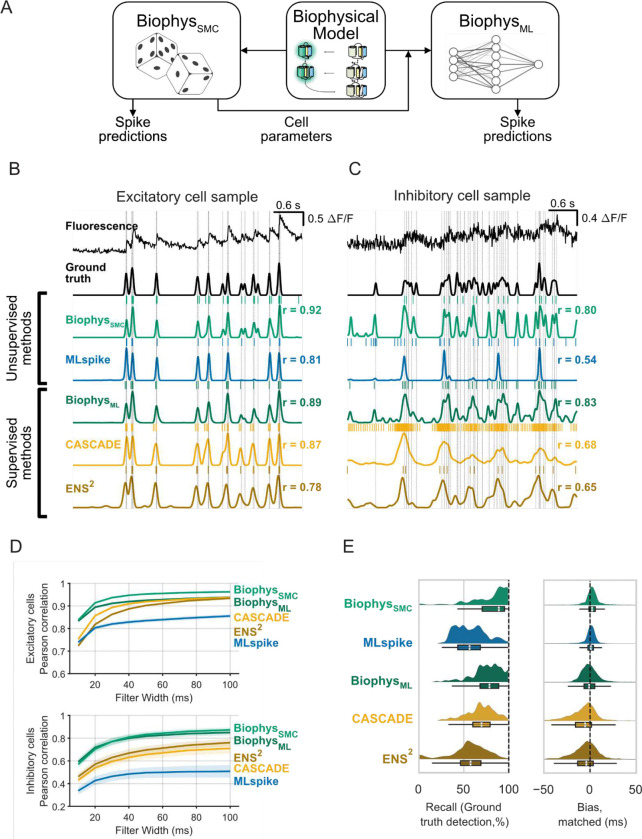
Improved action potential inference from generative model-based methods. (**A**) A generative model for imputing action potential times based on iterative fitting. The biophysical GCaMP model was held fixed while cell parameters were varied. Responses to action potential sequences were generated to fit cell parameters (Biophys_SMC_). After fitting was complete, the Biophys_SMC_ model was then used to train a machine learning based model. (**B, C**) Imputed spike probabilities using a variety of unsupervised and supervised models. Values of r indicate Pearson correlation between the time course of ground truth and that of the imputed probabilities for data filtered at 20 ms. Vertical lines indicate true spike times and tick marks indicate imputed spikes. (**B**) shows data from an excitatory cell while (**C**) shows data from an inhibitory cell. (**D**) Pearson correlations for different filtering time constants. (**E**) Discrete spike estimate statistics. Left panels shows the average time between the nearest imputed and ground truth spikes while the right shows the normalized number of spikes that were over or underestimated. Individual points are calculated over 8-second epochs covering 1000 data points randomly selected from the full dataset.(**E**) Timing and detection accuracy of the different imputation models. Left, distribution of per-spike timing error for the various methods. Smoothed histograms and box/whisker plots summarize all errors. Right, epoch-wise recall of the various methods indicating the percent of ground truth spikes recovered.

The Biophys_SMC_ approach to spike inference is computationally time-consuming due to the SMC sampling. Therefore we also used the GCaMP model to create a synthetic training dataset to train a machine learning algorithm (Rupprecht 2021). Specifically, we generated synthetic fluorescence data for training using cell parameters derived from the Biophys_SMC_ along with randomly generated spikes, noise, and baseline drift designed to mimic the statistics of the dataset targeted for inference (Figure 4A). In this way, we obtained two methods for spike inference: an unsupervised, generative Bayesian approach (Biophys_SMC_), and a computationally faster machine-learning approach (Biophys_ML_) which was generated by using the outputs of the unsupervised approach to train a supervised-learning neural network.

We benchmarked our spike inference methods against state-of-the-art supervised and unsupervised approaches for spike inference, using excitatory and inhibitory cell responses from the jGCaMP8f ground truth dataset (Zhang et al., 2023). We used 154 18-second epochs of firing in 37 excitatory neurons and 3 inhibitory neurons, containing 8,161 ground truth action potentials, with a median of 34 (IQR, 16–69) spikes per epoch. For a generative method comparison, we used MLspike, an established algorithm based on an alternative generative model of calcium-fluorescence dynamics (Deneux et al., 2016). For supervised-method comparisons, we used the CASCADE (Rupprecht et al., 2021) and ENS^2^ (Zhou et al., 2023) global-trained networks, which have been reported to outperform other methods on a variety of benchmark tests.

We first examined the ability of our methods to estimate continuous changes in activity. Imputed spike predictions and ground truth spikes were smoothed with Gaussian filters of various widths. Biophys_SMC_ and Biophys_ML_ model predictions were tightly locked to true spike patterns for both excitatory and inhibitory classes (Figure 4B, C), as indicated by the higher correlations between our method predictions and ground truth at all filter widths, particularly at ten and twenty milliseconds. Results with CASCADE were not improved by training the network on the jGCaMP8f dataset itself (Supplementary Figure 8).

We quantified the ability of various methods to track changes in firing using the Pearson correlation between continuous spike probability, filtered on various timescales, and the smoothed time course of ground truth (Figure 4D). For excitatory neurons (*n* = 142 epochs), a mixed effects model shows that correlation depends on the method used (method and filter width as fixed effects, *F*(4,4935) = 171.1, *p* = 2e-137). Method-wise comparisons demonstrate that Biophys_SMC_ outperforms MLspike (*p* = 6.5e-62, *d* = 1.1), CASCADE (*p* = 9.0e-47, *d* = 0.8), and ENS^2^ (*p* = 2.3e-82, *d* = 1.0). Likewise, Biophys_ML_ outperformed MLspike (*p* = 3.9e-56, *d* = 1.1), CASCADE (*p* = 9.8e-42, *d* = 0.3), and ENS^2^ (*p* = 9.4e-76, *d* = 0.6). Biophys_ML_ outperformed CASCADE at filter widths up to 30 milliseconds, ENS^2^ up to 76 milliseconds, and MLspike up to 100 milliseconds, the widest filter tested.

For the inhibitory cell dataset (*n* = 12 epochs), a similarly-structured mixed effect model again showed that correlation depends on method (*F*(4,385) = 14.7, *p* = 3.6e-11) with improved performance with Biphys_SMC_ (vs. MLspike *p* = 4.1e-9, *d* = 1.9; vs. CASCADE *p* = 3.3e-4, *d* = 1.0; vs. ENS^2^ *p* = 0.005, *d* = 1.1) and Biophys_ML_ (vs. MLspike *p* = 1.4e-10, *d* = 2.0; vs. CASCADE *p* = 3.3e-5, *d* = 1.2; vs. ENS^2^ *p* = 8.2e-4, *d* = 1.4). Together, these comparisons demonstrate the superior ability of Biophys_SMC_ and BiophysML to estimate continuous spike probability on a variety of timescales and in different cell types.

Next, we calculated how well the different methods were able to capture discrete spike times. First, we matched imputed spikes to the nearest ground-truth spike within a 10 ms interval to avoid double-counting of spikes and penalize imprecise imputed spikes (Supplementary Figure 9). Our biophysical methods consistently found ground truth events at a higher rate than other methods as assessed by sensitivity (also called recall, defined as true positives as a fraction of all ground truth events) (Figure 4E left, Table 1; repeated measures ANOVA (*F*(3,420) = 88.4, *p* = 2.25e-44), Biophys_SMC_ vs. MLspike *p* = 4.0e-9, *d* = 1.1; vs. CASCADE *p* = 4.0e-9, *d* = 0.6; vs. ENS^2^ *p* = 4.0e-9, *d* = 1.0; (*F*(3,420) = 81.6, *p* = 1.14e-41) Biophys_ML_ vs. MLspike *p* = 4.0e-9, *d* = 1.1; vs. CASCADE *p* = 4.0e-9, *d* = 0.7; vs. ENS^2^ *p* = 4.0e-9, *d* = 1.0).

We further tested the methods using a standard measure of predictive performance, the F-score. The F-score is the harmonic mean of precision (true positives as a fraction of all imputed events) and recall and has a maximum value of 1 thus allowing assessment of performance considering both false negatives as well as false positives. Taking this broader assessment into account, we found that the biophysical methods again outperform the other methods (Table 1; repeated measures ANOVA (*F*(3,420) = 11.7, *p* = 5.23e-7), Biophys_SMC_ vs. MLspike *p* = 3.8e-9, *d* = 1.1; vs. CASCADE *p* = 3.8e-9, *d* = 0.8; vs. ENS^2^ *p* = 3.8e-9, *d* = 0.9; (*F*(3,420) = 4.00, *p* = 0.008) Biophys_ML_ vs. MLspike *p* = 3.8e-9, *d* = 0.9; vs. CASCADE *p* = 3.8e-9, *d* = 0.6; vs. ENS^2^ *p* = 3.8e-9, *d* = 0.6). Thus, our biophysically-based methods provide unprecedented temporal accuracy and precision compared to current state-of-the-art approaches to spike predictions based on jGCaMP8f imaging datasets.

Finally, we tested the ability of our methods to estimate individual spike times. We first calculated the time between imputed spikes and the nearest matched ground-truth spike in a 100 ms interval to assess bias and uncertainty in spike time predictions (Figure 4E, right, Supplementary Figure 10). On an epoch-by-epoch basis, both of the supervised methods predicted spike times that tended to precede ground-truth times (CASCADE, 7.45 ms; ENS^2^, 7.67 ms); MLspike showed a smaller bias (4.93 ms). In contrast, both biophysical methods removed this bias (Table 1; repeated measures ANOVA, Biophys_SMC_ vs. MLspike *p* = 6.0e-8, *d* = 0.5; vs. CASCADE *p* = 6.0e-8, *d* = 0.8; vs. ENS^2^ *p* = 6.0e-8, *d* = 0.6; Biophys_ML_ vs. MLspike *p* = 1.8e-6, *d* = 0.4; vs. CASCADE *p* = 6.0e-8, *d* = 0.9; vs. ENS^2^ *p* = 1.8e-7, *d* = 0.5).

The biophysical methods also reported spike times with lower uncertainty (Biophys_SMC_ median 3.8, IQR 1.8–7.3 ms; Biophys_ML_ median 5.8, IQR 2.7–10.3 ms) than the other supervised methods (CASCADE median 8.2, IQR 3.5–18.2 ms; ENS^2^ median 8.3, IQR 3.8–16.6 ms) and superior epoch-wise statistics than all other methods (repeated measures ANOVA, Biophys_SMC_ vs. MLspike *p* = 0.01, *d* = 0.3; vs. CASCADE *p* = 6.0e-8, *d* = 0.5; vs. ENS^2^ *p* = 1.2e-7, *d* = 0.5; Biophys_ML_ vs. MLspike *p* = 0.4, *d* = 0.1; vs. CASCADE *p* = 6.0e-8, *d* = 0.5; vs. ENS^2^ *p* = 1.5e-5, *d* = 0.4). In summary, the generative biophysical model-based methods outperformed existing in-class and out-of-class alternatives, exceeding previous limits in the accuracy of GCaMP-based spike decoding.

## Discussion

In this study, we identified and characterized nonlinear distortions arising from spike activity patterns in fluorescence signals from three fast GCaMP variants, GCaMP6f, jGCaMP7f, and jGCaMP8f. We discovered a novel use-dependent slowing of the fluorescence decay during periods of sustained activity. Using these empirical observations, we constructed a biophysical model which served as the foundation of an unsupervised (Biophys_SMC_) and a supervised (Biophys_ML_) spike inference method. These biophysically-based inference methods extracted spikes with greater precision than existing methods. The advantage of biophysical model-based approaches was most apparent under high-bandwidth conditions.

Our spike-decoding framework was composed of three basic parts. First, we made recordings in a minimal tissue-based system (acute slices) where paired fluorescent/spike recordings were as easy as possible to make. Second, we used in vitro recordings where all time-varying parameters of experiments were known precisely, save the GCaMP response itself. Finally, we used these recordings to jointly constrain models of the GCaMP response per se and to suggest an appropriate generative model of the cellular response.

Our study clarifies how response nonlinearities can differ between indicators. Earlier GCaMPs (6f and 7f) showed a facilitation-like nonlinearity in their fluorescence response lasting tens of milliseconds, amplifying signals from closely timed spikes as previously noted (Éltes et al., 2019; Wei et al., 2020; Huang et al., 2021; Schoenfeld et al., 2021). We found an additional, novel nonlinearity: a persistent use-dependence that builds progressively during periods of heightened activity. This second nonlinearity was particularly apparent for jGCaMP8f, whose half-decay responses were slowed by up to three-fold. We showed that this effect is problematic for unsupervised methods that assume a linear mapping of spikes to fluorescence. In the linear SMC approach we used as a benchmark (PGBAR, (Diana et al., 2024)), elongated decay dynamics drove spurious predictions as the hysteretic effect forced the latent states to match the fluorescence dynamics (Figure 3G, H). This linear SMC, as well as MLspike (Deneux et al., 2016), were particularly poor spike predictors in the context of low SNR, high spike-rate data from inhibitory cells (Figure 3H; Figure 4C).

By leveraging a biophysically-based understanding of sensor behavior, we developed two complementary, high-performance spike inference methods. Initially, we applied the generative model directly within an unsupervised SMC framework to substantially improve accuracy, albeit with a computational cost inherent to Monte Carlo-based approaches (Vogelstein et al., 2010; Speiser et al., 2017). To address this limitation, we then used our biophysical model to produce large, synthetic datasets to train a supervised neural network that performed more quickly.

To our knowledge, our study is the first to report improvements in a machine learning training using a generative physical model approach. Because the biophysical model captures important features of GCaMP responses, we were able to use it as the engine for exploring how GCaMPs would react under different cellular and activity contexts. We achieved substantial improvements in spike time estimates by training our model on simulated spike patterns that explored a wide range of timing and patterns. The size and diversity of our training may explain why Biophys_ML_ outperformed CASCADE trained on the jGCaMP8f data itself. While large, the jGCaMP8f excitatory cell dataset still falls within a range over which increasing training set size would be expected to improve performance (Rupprecht et al., 2021; Zhou et al., 2023). Indeed, limited training data is a fundamental hurdle for any machine learning approach, a problem that is particularly acute in the context of labor-intensive datasets such as paired optical/electrophysiology considered here.

However, training of supervised neural networks, even on extensive, heterogeneous datasets, was insufficient to guarantee accurate inference for sensors with previously unseen properties. Both ENS^2^ and CASCADE, which rank highly on established benchmarks, exhibited temporal biases and overestimated rise times for jGCaMP8f (Figure 4). Notably, in contrast to a published report (Zhou et al., 2023), CASCADE outperformed ENS^2^ on all aspects of inference in the jGCaMP8f dataset. This result highlights that observations made with neural networks trained on broad datasets do not generalize in instances where indicator properties differ substantially from those in the training dataset. Taken together, these results suggest that for both current and future calcium indicator proteins, building an effective supervised inference approach requires a thorough exploration of relevant dynamic regimes using either labor-intensive ground truth or the generative approach we describe here.

In summary, this study elucidates the biophysical mechanisms underlying GECI fluorescence responses and offers practical solutions for more accurate and reliable neural activity detection. As a final note, our approach may serve as a generalizable roadmap for linking fluorescence measurements to underlying neural activity. For example, the steps we introduce here are likely to generalize to other GECI variants—including red-shifted (Inoue et al., 2015, 2019; Dana et al., 2016) and chemigenetic sensors (Deo et al., 2021; Farrants et al., 2024). Appropriate state and sensor models may allow extension to other genetically encoded sensors including those designed to detect neuromodulator release (e.g., Patriarchi et al., 2018; Sun et al., 2018) and voltage change (e.g., Evans et al., 2023; Platisa et al., 2023).

## Methods

### Animals.

The following strains were used for physiological experiments: C57Bl/6J (Jackson Laboratories stock 000664); CB6F1 (BalbC and C57Bl/6J F1); and Gabra6 (B6;129P2-Gabra6tm2(cre)Wwis/Mmucd). Both males and females were used and were between 40 and 130 days old. Animals were used in accordance with Princeton University’s Institutional Animal Care and Use Committee and Institut Pasteur’s CEEA-Paris1 approved protocols.

### Protein purification.

GCaMP variants were cloned into the pET28b protein expression vector using GCaMP6f, 7f, and 7s backbones generously provided by Douglas Kim (Janelia) and Addgene (104488). Plasmids were transformed into IPTG-inducible BL21(DE3) competent E. coli (MilliporeSigma 694504). Starter cultures of transformed cells were grown in 5 ml LB medium with 50 mg/L kanamycin at 37°C for 4 hours. Cultures were then added to 1 L LB medium with 50 mg/L kanamycin and grown at 37°C until the OD at 600 nm reached 1.0. 1 mM IPTG was added and the incubation temperature dropped to 25°C overnight for optimal protein expression.

Liquid cultures were centrifuged at 6,000 relative centrifugal force (rcf) for 10 minutes. Pellets were resuspended in 25 mM Tris-HCl, 500 mM NaCl, 20 mM imidazole, pH 8, with 1 mM PMSF for protease inhibition. Cells were mechanically lysed through an Emulsiflex homogenizer (ATA Scientific). Cell debris was removed through centrifugation at 13,000 rcf for 45 minutes at 4°C and the supernatant bound to nickel-NTA agarose (ThermoScientific 25215). Protein was eluted with 25 mM Tris-HCl, 500 mM NaCl, 500 mM imidazole, pH 8. Proteins were further concentrated using 10 kDa MWCO centrifugal filters (MilliporeSigma UFC901008) and desalted (GE Healthcare 17085101) into 130 mM KMOPS (in mM:100 KCl, 30 MOPS, pH 7.2), or a buffer mimicking intracellular conditions (in mM: 125 KCl, 14.5 KOH, 30 NaCl, 0.7 Mg2Cl, 10 HEPES) termed internal buffer as modified from (Tran et al., 2018). Protein concentrations were determined by absorption spectrophotometry using alkali denaturation in 0.1M NaOH. Under these conditions, mature GCaMP chromophore exhibits a standard molar absorption coefficient of 44,000M-1cm-1 at 447 nm, allowing direct recovery of GCaMP concentration using Beer’s law (Ward, 2005; Barnett et al., 2017).

### Protein steady-state and kinetic characterization.

Excitation and emission spectra were measured on a PTI Quantamaster 800 spectro-fluorometer (Horiba Jobin Yvon Inc., Edison, NJ, USA) with data collection under control of Felix GX software. For calcium-dependent fluorescence, steady-state measurements were made at 20°C using 0.15 μM of purified protein suspended in zero-Ca2+ buffer or high-Ca2+ buffer from a calcium calibration kit (ThermoFisher C3008MP). The zero-Ca2+ buffer was reciprocally diluted with the high-Ca2+ buffer to reach free Ca2+ concentrations between 0.01 and 10 μM. Hill coefficient and Kd were estimated by fitting steady state data to the Hill equation.

The fluorometer was also used to prepare reagents for stopped-flow kinetic measurements. Nominally Ca2+-free buffers included KMOPS or internal buffer supplemented by 2 mM BAPTA (Invitrogen B1204). Increasing amounts of 1 M CaCl2 were added to create free calcium steps of 0 nM, 50 nM, and increasing by approximately 2x free calcium in ten steps to 10 μM free calcium. Indicators were diluted to 175 nM in 0.1mM solution buffered by 0.1 mM BAPTA (see design considerations in Supplementary Figure 2).

Off-response kinetics buffers were designed to have a starting free calcium concentration of 10 μM in 100 μM BAPTA and a final nominal concentration of 0 nM or 50 nM free calcium when mixed to Ca2+-free buffer. Proteins were diluted to 35 nM in the 10 μM free calcium buffer. In all cases, fura-2 (Molecular Probes F6799) and fura-4F (Invitrogen F14174) were used to calibrate free calcium concentrations.

On-response and off-response kinetics were measured using an Applied Photophysics SX20 stopped-flow spectrometer. Excitation light was provided by a xenon arc lamp directed through a monochromator set to a 4 nm passband centered on 480 nm, while emissions were collected through a 550 nm longpass filter (Newport FR-OG550). Dead time in the range of 0.6–1.5 ms was estimated by extrapolation from measurements using OGB-1 (Life Technologies, O6806). Measurements were made at 37°C with 60 μl per reaction chamber.

### Adeno-associated virus constructs.

Adeno-associated viruses (AAVs) were prepared by cloning the GCaMP variants into pAAV backbones (GenScript, Piscataway, NJ). Plasmids were incorporated into AAV serotype 1 (AAV2/1) and serotype DJ (AAV DJ) by the Princeton Neuroscience Institute Viral Neuroengineering Laboratory.

### Stereotactic viral Injection.

Viral vectors were injected stereotactically into the cerebellar vermis. Mice were deeply anesthetized by intraperitoneal injections of 1.5% ketamine (Mérial) and 0.05% xylazine (Bayer). 2% Xylocaine (Newpharma) was applied to the cranial incision. 100 nl vermal (6.5 mm caudal to bregma, lateral 0.2 mm, ventral 3.6 mm and 3.4 mm) and 100 nl Crus I (6 mm caudal to bregma, lateral 3 mm, ventral 0.5 mm) injections were infused over one minute. To allow for optimal transgene expression, mice were imaged 2 to 4 weeks after injection.

### Slice preparation.

Animals were euthanized by rapid decapitation. The brains were quickly removed and placed in an ice-cold solution containing (in mM) 2.5 KCl, 0.5 CaCl2, 4 MgCl2, 1.25 NaH2PO4, 24 NaHCO3, 25 glucose, 230 sucrose, and 0.5 ascorbic acid. The buffer was bubbled with 95% O2 and 5% CO2. A Leica VT1200S vibratome was used to prepare coronal slices (200 μm), which were incubated at 32 °C for 30 min in (in mM): 85 NaCl, 2.5 KCl, 0.5 CaCl2, 4 MgCl2, 1.25 NaH2PO4, 24 NaHCO3, 25 glucose, 75 sucrose, and 0.5 ascorbic acid. The external recording solution contained (in mM) 125 NaCl, 2.5 KCl, 1.5 or 2.0 CaCl2, 1.5 or 1.0 MgCl2, 1.25 NaH2PO4, 25 NaHCO3, 25 glucose, and 0.5 ascorbic acid. Slices were maintained at room temperature for up to 6 h. All slice recordings were performed at 36–38°C.

### Imaging in cerebellar brain slices.

Parallel fiber boutons expressing fluorescent virus were imaged with an Ultima two-photon scanning scanhead (Bruker Nano Surfaces Division, Middleton, WI, USA) that was mounted on an Olympus BX61W1 microscope equipped with a water-immersion objective (60×/1.1-NA; Olympus Optical, Tokyo, Japan) and infrared Dodt-gradient contrast. Two-photon excitation was performed with a pulsed Ti:Sapphire laser (DeepSee, Spectra-Physics, France) tuned to 920 nm. Extracellular parallel fiber axonal stimulation was performed with a constant voltage stimulator (Digitimer Ltd, Letchworth Garden City, UK) and a patch pipette (typically with a tip resistance of 4–6 MΩ) filled with artificial cerebrospinal fluid and placed in the molecular layer adjacent to labeled parallel fibers. The fluorescence of single cerebellar granule cells was monitored using circular line scans along the cytoplasm of the GC somata or straight line scans across individual parallel fiber boutons. Typical line rates were ~1 kHz. To determine threshold fluorescence, extracellular stimulation intensity was increased until clear fluorescence responses were observed after a train of 20–60 60-μs pulses at 100 or 300 Hz. To ensure action potential initiation for each stimulus pulse, the stimulus voltage was increased by 5 V above threshold.

### Biophysical and cell models.

We modeled GCaMP fluorescence as arising from semi-independent (“hemi-independent”) (Lai et al., 2015) calcium binding at two lobes, each of which can subsequently bind a target peptide to become fluorescent. Formally, each lobe transitions between an unbound and a calcium-bound state, which is governed by first-order binding and unbinding reactions:

eqn. 1 $$\frac{d\left({\text{bound}}_{\text{lobe}}\right)}{dt}=k_{\text{on,lobe}}\;{unbound}_{\text{lobe}}-k_{\text{off,lobe}}\;{bound}_{\text{lobe}}$$

where $k_{\text{on,lobe}}$ and $k_{\text{off,lobe}}$ are rate constants for binding and unbinding at each lobe. We defined:

eqn. 2 $$k_{\text{on,lobe}}=\frac{k_{\text{off},\text{lobe}}}{{\left(K_{a,\text{lobe}}\right)}^{H_{\text{lobe}}}}$$

where $K_{a,\text{lobe}}$ is an apparent dissociation constant and $H_{\text{lobe}}$ is a Hill coefficient describing cooperative binding. Once a lobe is bound with calcium, it can transition to a fluorescent state by binding the target peptide. This step similarly follows first-order kinetics:

eqn. 3 $$\frac{d(\text{fluorescent})}{dt}=k_{\text{on,peptide,lobe}}{\text{bound}}_{\text{lobe}}-k_{\text{off,peptide,lobe}}\;\text{fluorescent}$$

All fluorescent states (i.e., those in which a lobe is calcium-bound and has the peptide bound) contribute equally to total fluorescence. For stopped-flow simulations, model fluorescence was normalized between 0 (occupancy of fluorescent states at zero calcium) and 1 (occupancy at saturating calcium). For cell-based simulations, fluorescence was scaled to a dimensionless quantity F by setting to 1 at 0 calcium and the parameter $R_{f}-$ the dynamic range of the indicator – at saturating calcium level. $\frac{\Delta F}{F}$ was then calculated as:

eqn. 4 $$\frac{\Delta F}{F}=\frac{F\left({Ca}_{\text{current}}\right)\;-\;F\left({Ca}_{basal}\right)}{F\left({Ca}_{basal}\right)}$$

where $F\left({Ca}_{\text{current}}\right)$ is the instantaneous normalized fluorescence at the current free-calcium level, and $F\left({Ca}_{basal}\right)$ is the steady-state fluorescence at basal calcium.

To simulate intracellular calcium transients (e.g., after action potentials), we extended the model from Rebola et al. 2019 (Rebola et al., 2019) by including an internal calcium reservoir with influx and efflux governed by Michaelis–Menten dynamics. Because cerebellar granule cells have high concentrations of fast, low affinity endogenous buffers, we linearized the buffer’s binding capacity $\kappa_{B}$ (Neher and Augustine, 1992) to reduce computational cost. Specifically, each small increment of free calcium ${Ca}_{\text{in}}$ was instantly equilibrated with endogenous buffer:

eqn. 5 $$\frac{d(BCa)}{dt}=\frac{{Ca}_{in}\;\kappa_{B}}{\kappa_{B}\;+\;1}$$

where BCa is the portion of endogenous buffer bound to calcium, a ratio of approximately 40 for our standard granule cell model. This linearization preserves the overall calcium balance while allowing faster numerical integration of high buffer concentration simulations.

For obtaining full GCaMP model parameterization we globally fit all traces obtained from stopped flow for each variant by gradient descent from 1500 quasi-randomly selected initial points in parameter space. GCaMP parameters from each run were then fixed and best fits were obtained across our ex vivo datasets by gradient descent over cell-specific parameters. The GCaMP model parameters which minimized errors across experimental contexts were then selected and fixed for each GCaMP variant.

### Bayesian and machine-learning spike inference algorithms.

In order to infer spike times from fluorescence time series using the biophysical model of jGCaMP8f described in the main text we adopted the Bayesian framework developed by Diana et al, 2024 based on sequential Monte Carlo methods (particle Gibbs with ancestor sampling, PGAS; (Lindsten et al., 2014; Diana et al., 2024) which enabled us to estimate simultaneously the posterior distributions of spike times and the parameters of the cell model, including the GCaMP concentration, calcium extrusion rates, peak amplitude of the calcium flux due to single action potentials and the dynamic range of the probe. Single point estimates of model parameters and spike times were obtained by using maximum-a-posteriori estimates.

We compared the performance of our method with MLSpike (Deneux et al., 2016) which employs a different model for the calcium indicator dynamics and baseline fluorescence. Spike inference in MLspike is carried out by discretizing all continuous dynamical variables in the model and applying the Viterbi algorithm to extract maximum-a-posteriori estimates of spike times. One of the weaknesses of this approach is the difficulty of estimating the model parameters, for which Deneux et al provide ad hoc calibration schemes. We directly addressed this issue using the PGAS sampling algorithm.

We compared our method with state-of-the-art supervised machine-learning models: CASCADE (Rupprecht et al., 2021) and ENS^2^ (Zhou et al., 2023). The two approaches use different network architectures. CASCADE returns the spike probability at each time point by taking as input a local time window by using a trained convolutional network while ENS^2^ uses the U-net applied on fixed-length segments of the original calcium trace.

### Analysis of discrete action potential imputation.

Continuous prediction traces were converted to discrete imputed spike times using the approach indicated by each published method. For Biophys_SMC_, spike predictions were calculated using the maximum a-posteriori of the posterior distribution of the spike state over time. For Biophys_ML_, spike predictions were calculated by optimally fitting a set of Gaussian kernels with prior width and height determined by approximating the smoothing filter applied to ground truth for model training as in (Rupprecht et al., 2021).

To avoid double-counting of imputed spikes for a given ground truth spike, we employed the linear assignment algorithm to identify unique imputed/ground truth pairs. We used the matlab function matchpairs at a cost of unassignment as specified in the main text to constrain time interval between such pairs as specified in the main text.True positives were then defined as imputed spikes with unique ground truth matches, with unmatched ground truth spikes giving the false negatives, and unmatched imputed spikes being false positives. Total true positives, false positives, and ground-truth spikes within epochs for each cell to form the distributions for F-score, precision, and recall/specificity.

For each epoch of ground-truth recording, the bias in each imputed spike time was calculated as the interval to the nearest ground-truth action potential while uncertainty was defined as the absolute value of this quantity. The measures were taken to either the nearest ground truth spike using a nearest neighbors search (unmatched) or to the nearest matched spike.

### Statistical presentation.

Box and whisker plots comprise a box indicating the median and 25–75th percentile range and whiskers indicating the shorter of 1.5 times the interquartile range or the extreme data point. Descriptive statistics were calculated using median-based methods, and the estimated standard deviation was defined as the median absolute deviation divided by 0.6745.

## Supplementary Material

Supplement 1

## Figures and Tables

**Table 1.** Single-spike classification metrics for calcium-based decoding methods. Median performance across epochs for a database of 37 excitatory cells with ground-truth electrical recordings and jGCaMP8f fluorescence signals. Values reported as median (IQR).

	Precision	Recall	F-Score	Accuracy	Mean bias (ms)	Uncertainty (ms)

Biophys_SMC_	0.84 (0.75, 0.92)	0.88 (0.71. 0.95)	0.83 (0.71, 0.91)	0.71 (0.55, 0.83)	1.9 (−2.9, 3.0)	3.8 (1.8, 7.3)
MLspike	0.70 (0.53, 0.93)	0.56 (0.43, 0.68)	0.60 (0.51, 0.70)	0.43 (0.34, 0.54)	−2.0 (−0.6. 0.9)	3.2 (1.5. 6.2)
Biophys_ML_	0.76 (0.68, 0.84)	0.78 (0.68, 0.89)	0.75 (0.70, 0.80)	0.61 (0.54, 0.67)	0.0 (−2.9, 3.0)	5.8 (2.7, 10.3)
CASCADE	0.69 (0.63, 0.78)	0.70 (0.60. 0.79)	0.69 (0.61, 0.74)	0.52 (0.45, 0.59)	−7.4 (−11.2, −3.2)	8.2 (3.5, 1.8.2)
ENS^2^	0.71 (0.65, 0.86)	0.57 (0.46. 0.69)	0.65 (0.56, 0.74)	0.48 (0.39, 0.58)	−6.6 (−12.3, −2.3)	8.3 (3.8, 16.5)
