# Supplementary material for: Precise calcium-to-spike inference using biophysical generative models
Source: Res Sq. 2025 Apr 24:rs.3.rs-6017950. Preprint. [Version 1] doi: 10.21203/rs.3.rs-6017950/v1 (PMC12045362; doi:10.21203/rs.3.rs-6017950/v1)
Supplement: Supplement 1 [file NIHPPRS6017950V1-supplement-1.pdf]

Supplementary Figure 1: jGCaMP8f response decay is captured by two kinetic components.

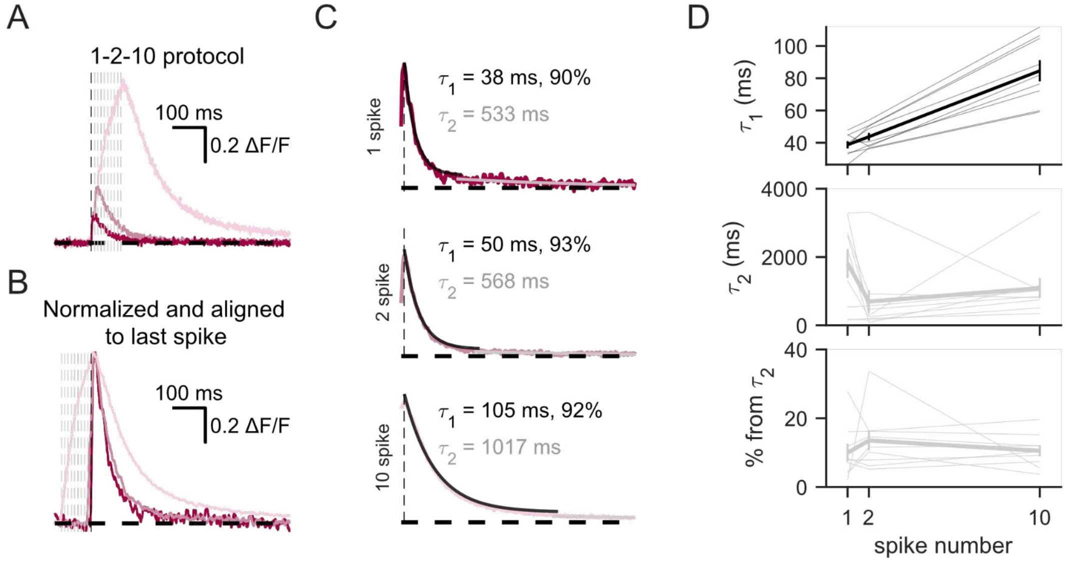

*(**A**) Representative single-bouton responses to 1, 2, and 10 stimuli overlaid and aligned by first stimulus time. (**B**) Responses normalized and aligned to last spike. (**C**) Example fits to dual sums of exponentials showing fast and slow components of decay. (**D**) Dependence of fast (top) and slow (middle) time constants on the number of stimuli, and relative contribution of the slow component (τ_2) to the overall time course (bottom). Individual bouton values shown as low linewidth traces with group means shown as thicker line and standard error of the mean as error bars, n = 6 boutons and 3 granule cell somata.*

Supplementary Figure 2: The impact of calcium clamp design on observed stopped-flow dynamics.

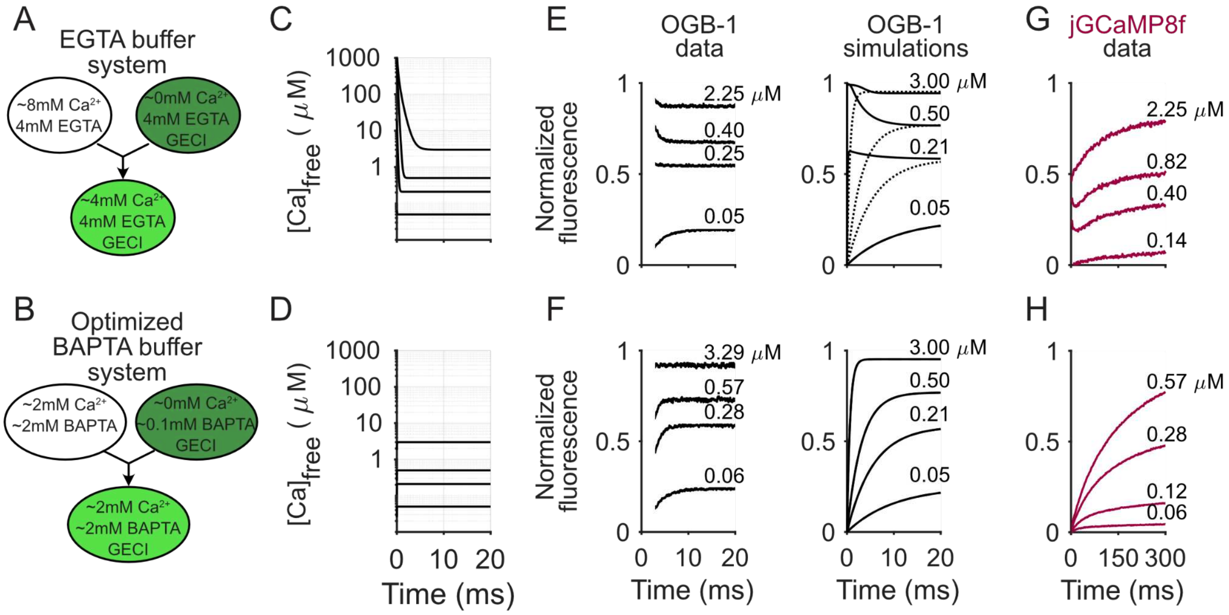

*(**A-B**) Schematic of stopped-flow buffer systems using (**A**) a slow buffer (EGTA) and containing unbuffered calcium in the calcium-containing bolus (**A**) and (**B**) a system with a fast buffer (BAPTA) and fully buffered calcium in the calcium-containing bolus. (**C**) Simulations of the EGTA-based system presented in (**A**) show uncontrolled free calcium concentrations in the first few milliseconds of large calcium steps, but (**D**) BAPTA-based experiments do not. (**E**) Measurements of OGB-1 responses (left) reflect uncontrolled responses that are matched by simulations of the buffer system (right). (**F**) OGB-1 data and simulations in BAPTA-based buffers. (**G**) Artifactual fast on-response to jGCaMP8f in EGTA-based buffer system. (**H**) True jGCaMP8f response in BAPTA-based buffer system.*

Supplementary Figure 3: Magnesium affects the properties of jGCaMP8f much more than older GCaMPs.

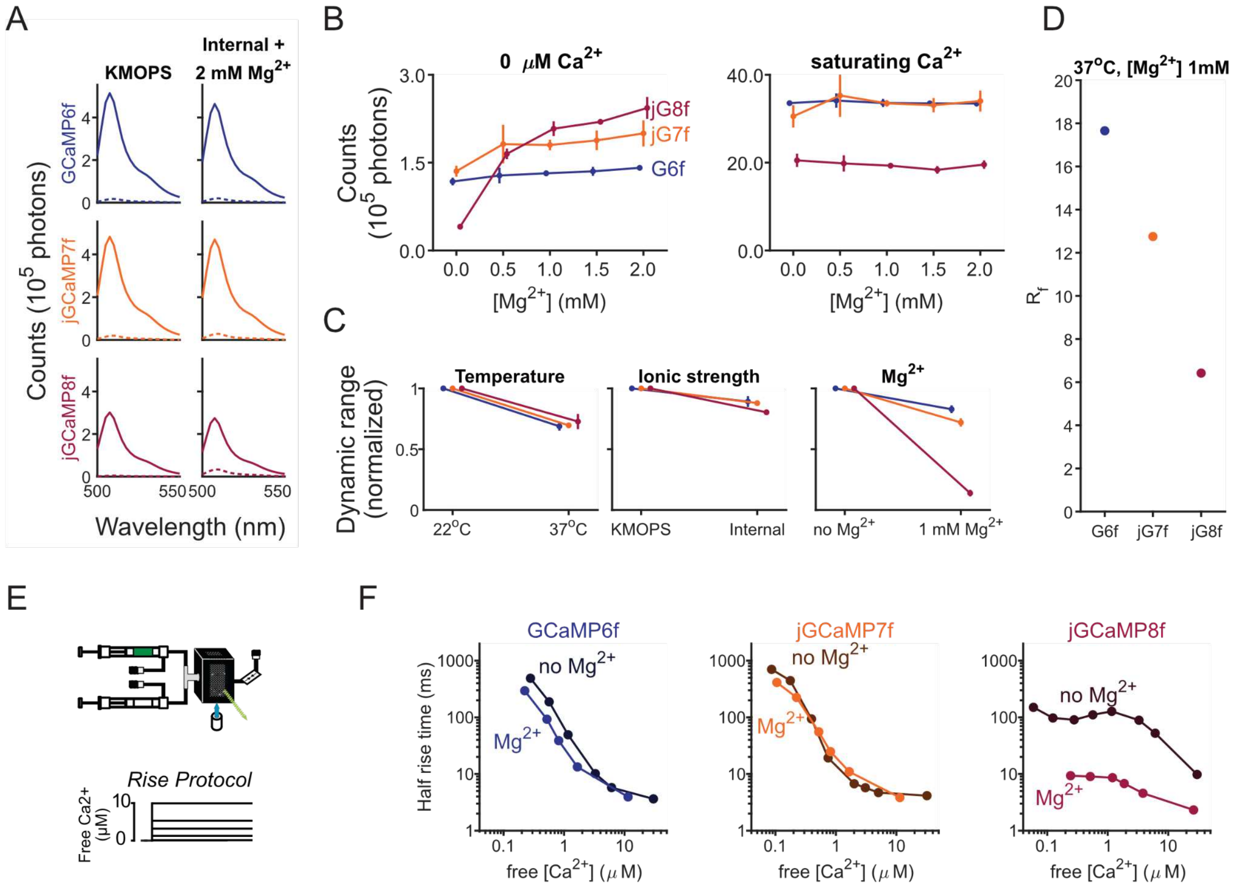

(***A***) *Emission spectra of GCaMP variants in standard protein purification buffer (KMOPS, left) and under intracellular conditions that include magnesium (2 mM Mg^{2+}). (**B**) Fluorimeter counts at 490 nm under calcium-free (left) or saturating calcium (right) conditions. jGCaMP8f, but not other variants, showed strong magnesium-dependence of fluorescence emission under low-calcium conditions. No indicator showed this effect at saturating calcium. (**C**) Quantification of the weak dependence of GECI dynamic range R_f (saturating calcium divided by calcium-free fluorescence output) on temperature (left) or ionic strength (center) compared to the effect seen with magnesium (right). (**D**) Dynamic range of GCaMP variants at mammalian near-physiological conditions. (**E**) Schematic of stopped-flow experiment to measure responses to rising steps of calcium concentration. (**F**) Half-rise times to calcium steps were accelerated most by magnesium for jGCaMP8f, less so for GCaMP6f, and not at all for jGCaMP7f. Error bars indicate standard error of the mean.*

Supplementary Figure 4: Biphasic responses of GCaMPs to increasing calcium steps.

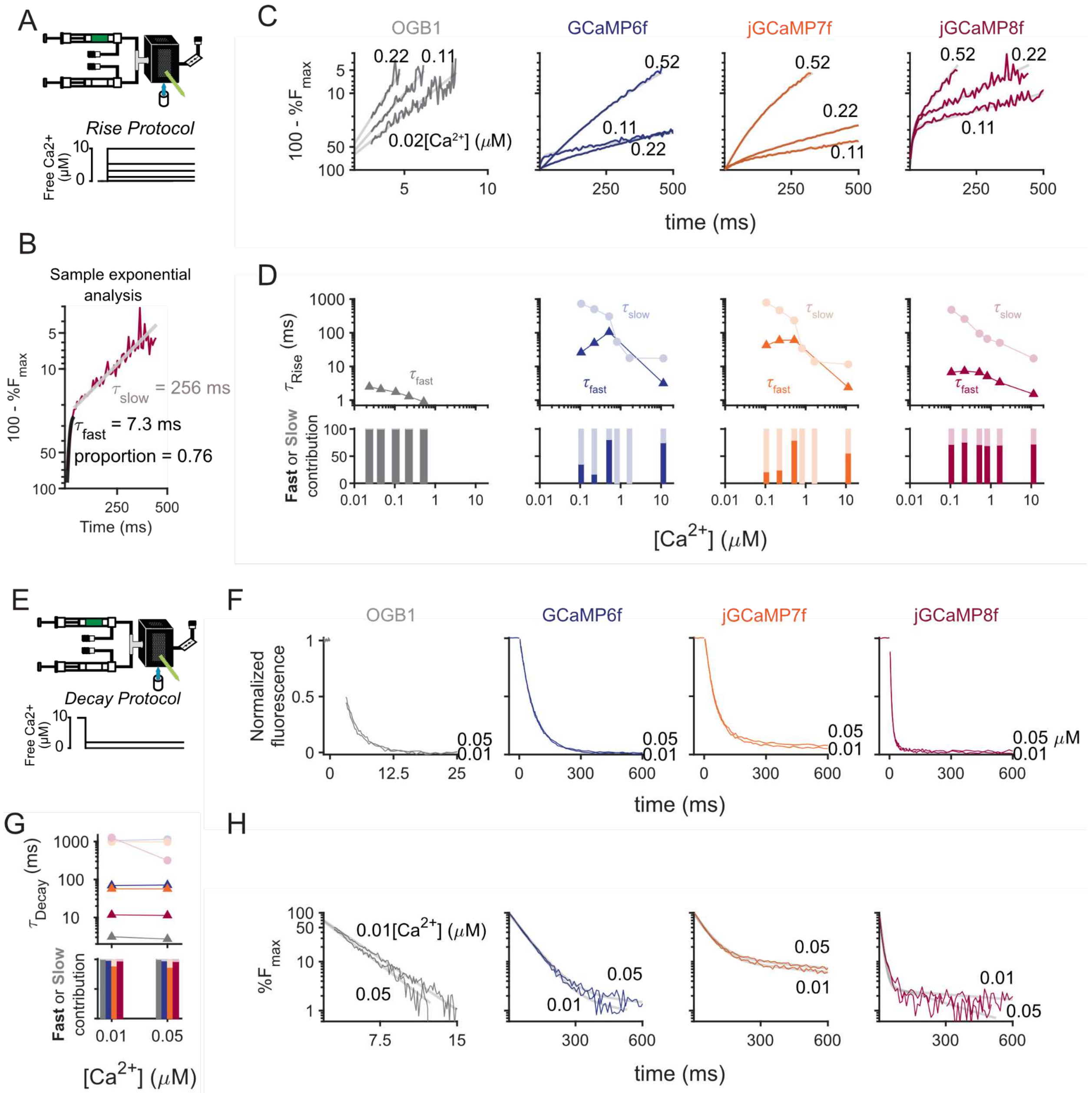

(***A***) *Schematic of stopped-flow experiment and rising calcium-step protocol.* (***B***) *Example analysis of fit to a sum of two exponentials.* (***C***) *Monoexponential rising kinetics for Oregon Green BAPTA-1 (OGB-1) and biexponential kinetics for GCaMP variants.* (***D***) *Dependence of fast and slow components on amplitude of calcium step. Upper plots show rate constants represented in the response at the indicated step. In the lower plots, vertical bars show relative amplitude of fast (dark) and slow (light) components.* (***E***) *Schematic of decreasing-calcium step*

*protocol. (**F**) Fluorescence responses to decreasing calcium steps. (**G**) Breakdown of kinetic components contributing to the decay of fluorescence as in (**D**). Due to the low number of steps considered, analysis for all variants is included in this single plot. (**H**) Monoexponential decay for OGB-1 and more complex responses for GCaMP variants.*

Supplementary Figure 5: Selection of GCaMP biophysical parameters using in vitro and ex vivo datasets.

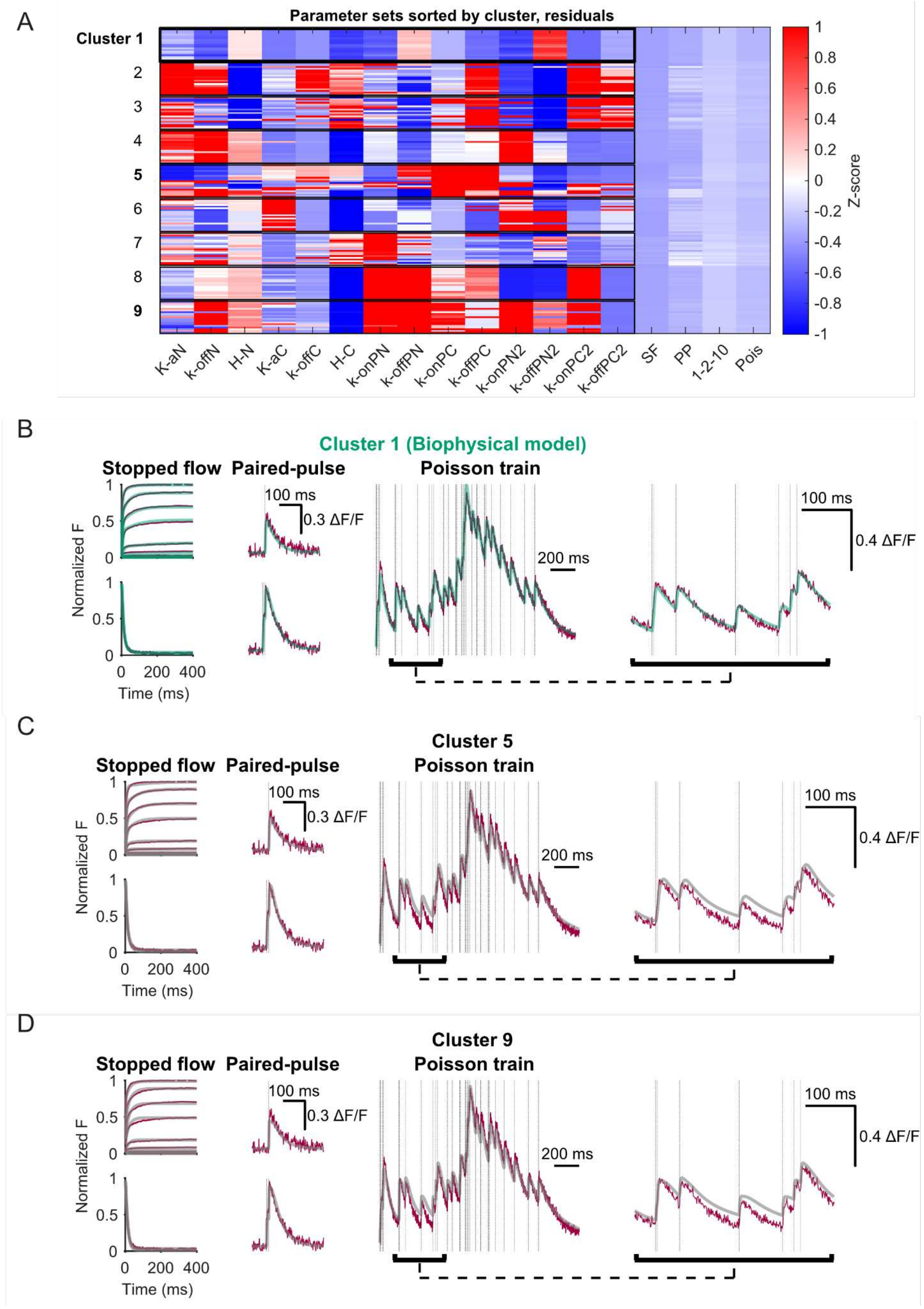

(***A***) *Distributions of parameters on fits from randomized seeds with GCaMP model parameters at figure left and residuals from best fits to stopped-flow and ex vivo experiments (paired pulse,*

*1-2-10 pulse, and Poisson train) based on optimizing cell parameters. GCaMP parameter sets were sorted by hierarchical clustering and then by lowest summed residuals within each cluster. This procedure allowed qualitative assessment of parameters that lay in different local minima of the parameter space. (**B**) Fitted data examples for cluster 1, which was used for all further modeling. (**C**) Fitted data for cluster 5. (**D**) Fitted data for cluster 9. In paired-pulse and Poisson train data, vertical lines indicate the times of action potentials. Expanded timescale of Poisson responses at right. Neither cluster 5 or 9 captured the differential slowing across the Poisson responses.*

Supplementary Figure 6: A full biophysical state analysis of GCaMP response.

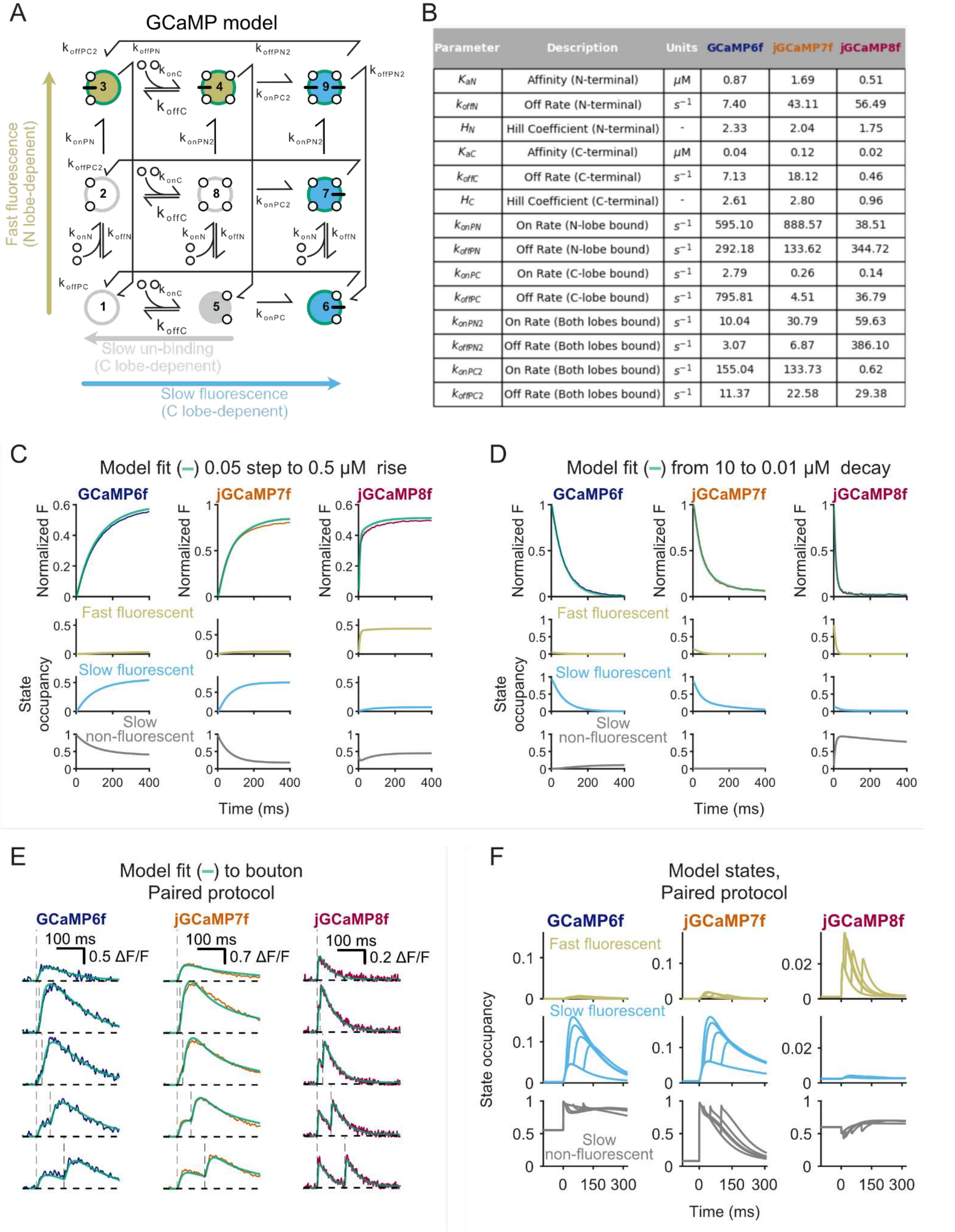

Parameter	Description	Units	GCaMP6f	jGCaMP7f	jGCaMP8f
K_{aN}	Affinity (N-terminal)	μM	0.87	1.69	0.51
k_{offN}	Off Rate (N-terminal)	s^{-1}	7.40	43.11	56.49
H_N	Hill Coefficient (N-terminal)	-	2.33	2.04	1.75
K_{aC}	Affinity (C-terminal)	μM	0.04	0.12	0.02
k_{offC}	Off Rate (C-terminal)	s^{-1}	7.13	18.12	0.46
H_C	Hill Coefficient (C-terminal)	-	2.61	2.80	0.96
k_{onPN}	On Rate (N-lobe bound)	s^{-1}	595.10	888.57	38.51
k_{offPN}	Off Rate (N-lobe bound)	s^{-1}	292.18	133.62	344.72
k_{onPC}	On Rate (C-lobe bound)	s^{-1}	2.79	0.26	0.14
k_{offPC}	Off Rate (C-lobe bound)	s^{-1}	795.81	4.51	36.79
k_{onPN2}	On Rate (Both lobes bound)	s^{-1}	10.04	30.79	59.63
k_{offPN2}	Off Rate (Both lobes bound)	s^{-1}	3.07	6.87	386.10
k_{onPC2}	On Rate (Both lobes bound)	s^{-1}	155.04	133.73	0.62
k_{offPC2}	Off Rate (Both lobes bound)	s^{-1}	11.37	22.58	29.38

*(**A**) Full biophysical state model of GCaMP response. Specific states referred to as "fast fluorescent", "slow fluorescent", and "slow, non-fluorescent" have centers color-coded as in model state traces below. (**B**) Best-fit parameter values for GCaMP6f, jGCaMP7f, and jGCaMP8f. (**C**) Model fits to 0.5 μM increasing step of calcium (top), with model state broken*

*down by occupancy. Note that jGCaMP8f is distinguished by its near-exclusive use of the fast fluorescent states. (**D**) As in (**C**) for decreasing step. jGCaMP8f is distinguished by fast, persistent entry into the slow, non-fluorescent state. (**E**) Model fits to fluorescence responses in presynaptic boutons of granule cells. (**F**) State occupancy for the fluorescence responses shown in (**E**). Fast responses arise in part from high occupancy of a slow, non-fluorescent state at basal calcium for jGCaMP8f which efficiently transitions to fluorescence on elevation of calcium.*

Supplementary Figure 7: Generative cell-based model of fluorescence time series

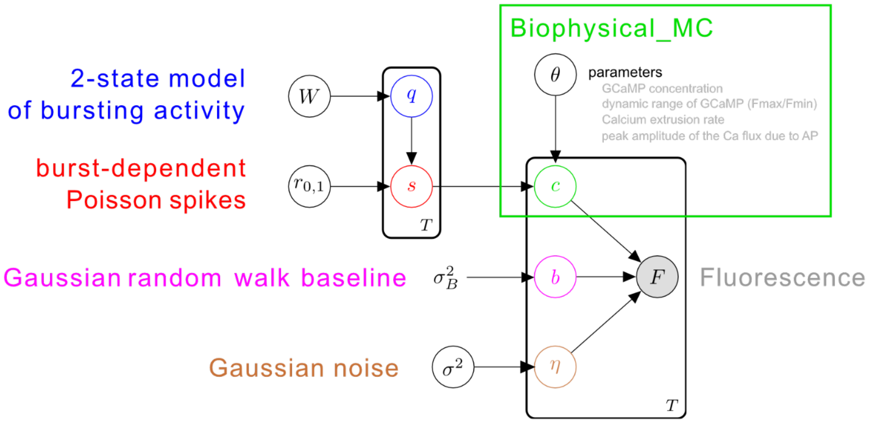

Graphical representation of the generative SMC model described in the main text. White circles denote unknown variables, gray circles denote measurements and bare variables are fixed prior hyperparameters. Plates denote groups of variables. Note that GCaMP model parameters are included, but fixed for the SMC approach.

Supplementary Figure 8: Training on jGCaMP8f data does not improve CASCADE performance.

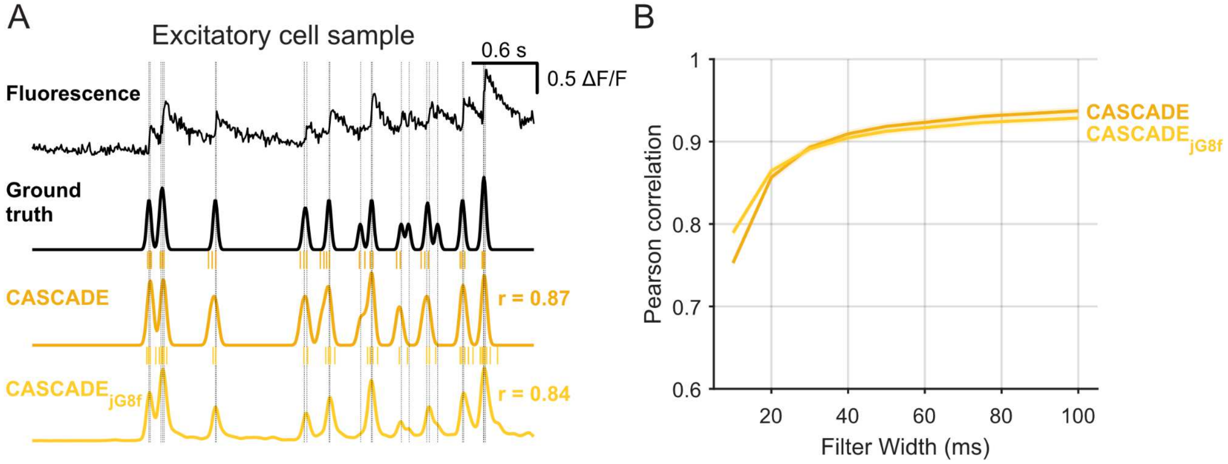

*(**A**) Imputed spike probabilities using the CASCADE Global Exc model or one trained on the jGCaMP8f dataset itself. Values of r indicate Pearson correlation between the time course of ground truth and that of the imputed probabilities for data filtered at 20 ms. Vertical lines indicate true spike times and tick marks indicate imputed spikes. (**B**) Pearson correlations for different filtering time constants.*

Supplementary Figure 9: Statistics of discrete spike estimate comparisons to ground truth spike times

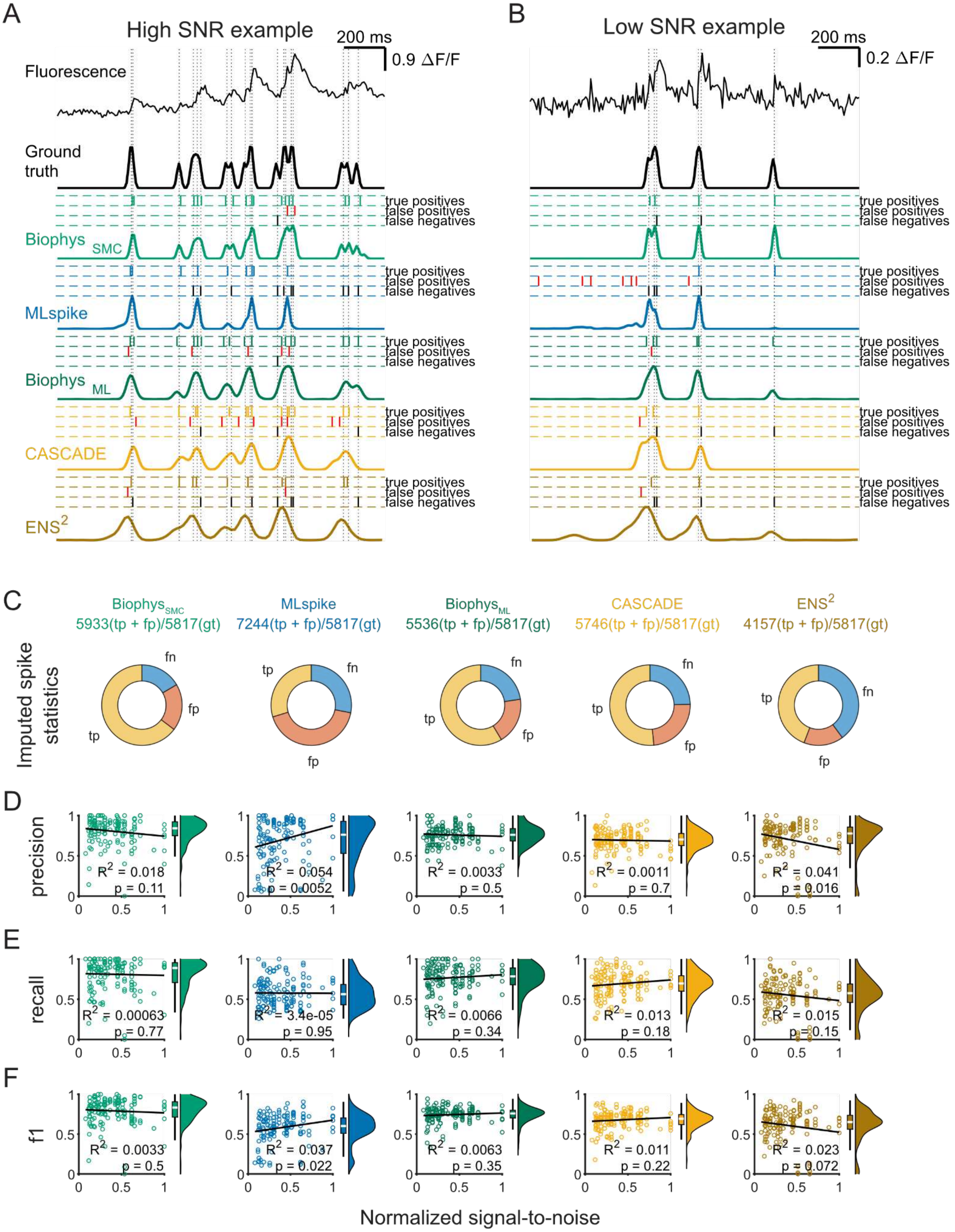

(***A, B***) *Continuous and discrete spike predictions across methods. The top two traces (black) show raw fluorescence and Gaussian-convolved (10 ms) ground truth spike times. Ground truth*

*spike times are indicated as vertical tick marks. Discrete imputed spikes for each method are shown above the corresponding continuous prediction trace, sorted into true positives, false positives, and false negatives. An epoch was selected from a representative cell which produced high signal-to-noise ratio (SNR) fluorescence changes in response to an action potential (**A**) or low SNR (**B**). (**C**) Columns show the proportions of imputed spikes that were scored as true positive (tp), false positive (fp), or false negative (fn) for each method with a comparison of all imputed spikes produced by each method (tp + fp) compared to total number of ground truth (gt) spikes from the 37 putative excitatory cells in the Janelia dataset. (**D-F**) Precision (tp/(tp + fn), **D**), Recall (tp/(tp + fp), **E**), and F-score (harmonic mean of recall and precision, **F**) as a function of the SNR of each recorded epoch. The black line in each plot indicates a regression fit to the data with r^2 indicating the amount of variation explained by SNR and p indicating significance of the t-statistic comparing a linear to a constant model. Of all methods, only MLspike shows reduced performance at the SNR levels tested across this dataset. In particular, the precision (propensity to find false negatives) was increased in low SNR conditions.*

Supplementary Figure 10: Comparison of matched and unmatched temporal differences across methods.

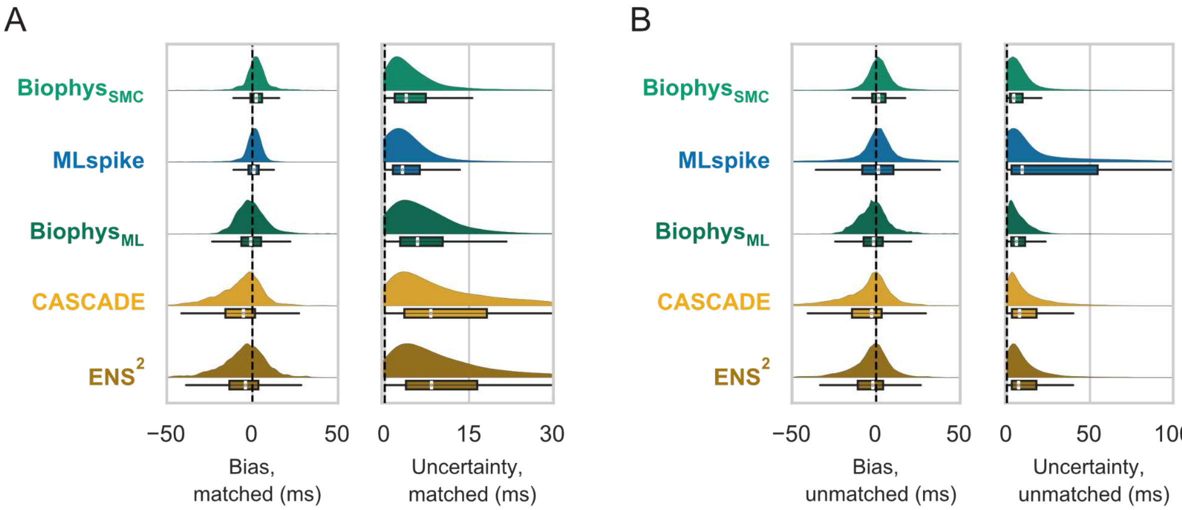

*(**A**) Bias and uncertainty between imputed and ground truth spikes matched with a 100 ms. Each plot is a kernel density estimate over a boxplot of indicated metric for each imputed spike. (**B**) As **A** for all imputed spikes compared to the nearest ground truth spike. Note that the supervised methods show increased bias and uncertainty when removing double-counted imputed spikes by using matched statistics.*
